# Prospective Validation of ATA Risk Score for Papillary Thyroid Microcarcinoma: An ITCO Real-World Study

**DOI:** 10.1210/clinem/dgaf190

**Published:** 2025-04-01

**Authors:** Simone De Leo, Giulia Brigante, Silvia D’Elia, Simona Censi, Bruno Madeo, Silvia Morelli, Alice Nervo, Andrea Repaci, Clotilde Sparano, Ilaria Stramazzo, Camilla Virili, Francesco Bertagna, Francesco Dondi, Efisio Puxeddu, Edoardo Talpacci, Maria Chiara Zatelli, Maria Rosaria Ambrosio, Francesco Felicetti, Alessandro Piovesan, Luisa Petrone, Virginia Adornato, Matteo Trevisan, Laura Fugazzola, Chiara Mele, Marco Zavattaro, Erica Solaroli, Nicola Salituro, Mattia Rossi, Loredana Pagano, Alessandra Colapinto, Cristina Basso, Graziano Ceresini, Michela Marina, Umberto Crocetti, Michela Massa, Maurilio Deandrea, Francesca Retta, Giovanna Spiazzi, Nicoletta Rolli, Rocco Bruno, Antonella Carbone, Mario Rotondi, Flavia Magri, Poupak Fallahi, Maria Grazia Chiofalo, Maria Giulia Santaguida, Salvatore Monti, Tommaso Porcelli, Roberto Castello, Alfonso Sagnella, Cristina Clausi, Giulia Di Dalmazi, Dario Tumino, Andrea Palermo, Antonio Brunetti, Andrea Lania, Andrea Liverani, Cosimo Durante, Umberto Ferraro Petrillo, Marco Alfo’, Sebastiano Filetti, Giorgio Grani

**Affiliations:** Endocrine Oncology Unit, Department of Endocrine and Metabolic Diseases, IRCCS Istituto Auxologico Italiano, Milan 20149, Italy; Unit of Endocrinology, Department of Biomedical, Metabolic and Neural Sciences, University of Modena and Reggio Emilia, Modena 41125, Italy; Department of Statistical Sciences, Sapienza University of Rome, Rome 00185, Italy; Operative Unit of Endocrinology Department of Medicine-DIMED University of Padua, Padua 35128, Italy; Unit of Endocrinology, Department of Biomedical, Metabolic and Neural Sciences, University of Modena and Reggio Emilia, Modena 41125, Italy; Department of Medicine and Surgery, University of Perugia, Perugia 06129, Italy; Oncological Endocrinology Unit, Department of Medical Sciences, Città della Salute e della Scienza Hospital, University of Turin, Turin 10126, Italy; Division of Endocrinology and Diabetes Prevention and Care, IRCCS Azienda Ospedaliero-Universitaria di Bologna, Bologna 40138, Italy; Endocrinology Unit, Department of Experimental and Clinical Biomedical Sciences “Mario Serio,” University of Florence, Florence 50134, Italy; Endocrinology Unit, Department of Medico-Surgical Sciences and Biotechnologies, Sapienza University of Rome, Latina 04100, Italy; Endocrinology Unit, Department of Medico-Surgical Sciences and Biotechnologies, Sapienza University of Rome, Latina 04100, Italy; Nuclear Medicine, University of Brescia and Spedali Civili di Brescia, Brescia 25123, Italy; Nuclear Medicine, University of Brescia and Spedali Civili di Brescia, Brescia 25123, Italy; Department of Medicine and Surgery, University of Perugia, Perugia 06129, Italy; Department of Medicine and Surgery, University of Perugia, Perugia 06129, Italy; Section of Endocrinology, Geriatrics and Internal Medicine, Department of Medical Sciences, University of Ferrara, Ferrara 44121, Italy; Section of Endocrinology, Geriatrics and Internal Medicine, Department of Medical Sciences, University of Ferrara, Ferrara 44121, Italy; Oncological Endocrinology Unit, Department of Medical Sciences, Città della Salute e della Scienza Hospital, University of Turin, Turin 10126, Italy; Oncological Endocrinology Unit, Department of Medical Sciences, Città della Salute e della Scienza Hospital, University of Turin, Turin 10126, Italy; Endocrinology Unit, Department of Experimental and Clinical Biomedical Sciences “Mario Serio,” University of Florence, Florence 50134, Italy; Endocrinology Unit, Department of Experimental and Clinical Biomedical Sciences “Mario Serio,” University of Florence, Florence 50134, Italy; Endocrine Oncology Unit, Department of Endocrine and Metabolic Diseases, IRCCS Istituto Auxologico Italiano, Milan 20149, Italy; Endocrine Oncology Unit, Department of Endocrine and Metabolic Diseases, IRCCS Istituto Auxologico Italiano, Milan 20149, Italy; Endocrinology, Department of Translational Medicine, University of Piemonte Orientale, Novara 28100, Italy; Endocrinology, Department of Translational Medicine, University of Piemonte Orientale, Novara 28100, Italy; Unit of Endocrinology, Department of Medicine, AUSL Bologna, Bologna 40124, Italy; Unit of Endocrinology, Department of Medicine, AUSL Bologna, Bologna 40124, Italy; Endocrinology, Diabetology and Metabolism, Department of Medical Sciences, University of Turin, Turin 10126, Italy; Endocrinology, Diabetology and Metabolism, Department of Medical Sciences, University of Turin, Turin 10126, Italy; Division of Endocrinology and Diabetes Prevention and Care, IRCCS Azienda Ospedaliero-Universitaria di Bologna, Bologna 40138, Italy; Division of Endocrinology and Diabetes Prevention and Care, IRCCS Azienda Ospedaliero-Universitaria di Bologna, Bologna 40138, Italy; Unit of Internal Medicine and Oncological Endocrinology, University Hospital of Parma, Parma 43126, Italy; Unit of Internal Medicine and Oncological Endocrinology, University Hospital of Parma, Parma 43126, Italy; Department of Medical Sciences, Hospital “Casa Sollievo della Sofferenza,” IRCCS, San Giovanni Rotondo 71013, Italy; Department of Medical Sciences, Hospital “Casa Sollievo della Sofferenza,” IRCCS, San Giovanni Rotondo 71013, Italy; UO Endocrinologia, Diabetologia e Malattie del metabolismo, AO Ordine Mauriziano, Torino 101128, Italy; UO Endocrinologia, Diabetologia e Malattie del metabolismo, AO Ordine Mauriziano, Torino 101128, Italy; Endocrinology and Diabetology Unit, Department of Medicine, Azienda Ospedaliera-Universitaria di Verona, Verona 37126, Italy; Endocrinology and Diabetology Unit, Department of Medicine, Azienda Ospedaliera-Universitaria di Verona, Verona 37126, Italy; Endocrine Unit, Tinchi Hospital, Asm Matera, Matera 75100, Italy; Endocrine Unit, Tinchi Hospital, Asm Matera, Matera 75100, Italy; Istituti Clinici Scientifici Maugeri IRCCS, Unit of Endocrinology and Metabolism and Department of Internal Medicine and Therapeutics, University of Pavia, Pavia 27100, Italy; Istituti Clinici Scientifici Maugeri IRCCS, Unit of Endocrinology and Metabolism and Department of Internal Medicine and Therapeutics, University of Pavia, Pavia 27100, Italy; Department of Surgery, Medical and Molecular Pathology and Critical Area, University of Pisa, Pisa 56100, Italy; Department Head and Neck, Istituto Nazionale Tumori, IRCCS Fondazione G. Pascale, Naples 80131, Italy; Endocrinology Unit, Department of Medico-Surgical Sciences and Biotechnologies, Sapienza University of Rome, Latina 04100, Italy; Endocrinology and Diabetes Unit, Azienda Ospedaliero-Universitaria Sant’Andrea, Sapienza University of Rome, Rome 00185, Italy; Department of Public Health, University of Naples “Federico II,” Naples 80138, Italy; Department of Medicine, Hospital and University of Verona, Verona 37134, Italy; Department of Medicine, Surgery and Neuroscience, Section of Endocrinology, University of Siena, Siena 53100, Italy; Operative Unit of Endocrinology Department of Medicine-DIMED University of Padua, Padua 35128, Italy; Department of Medicine and Aging Sciences, University “G. d'Annunzio” of Chieti-Pescara, Chieti 66100, Italy; Department of Clinical and Experimental Medicine, University of Catania, Catania 95122, Italy; Unit of Metabolic Bone and Thyroid Diseases, Fondazione Policlinico Campus Bio-Medico and Endocrinology and Diabetes Campus Bio-Medico University, Rome 00128, Italy; Department of Health Sciences, University of “Magna Græcia,” Catanzaro 88100, Italy; Department of Biomedical Sciences, Humanitas University, and Endocrinology, Diabetology and Andrology Unit, IRCCS Humanitas Research Hospital, Rozzano, Milan 20089, Italy; Department of Surgery, Regina Apostolorum Hospital, Albano Laziale, Rome 00041, Italy; Dept. of Translational and Precision Medicine, Sapienza University of Rome, Rome 00161, Italy; Department of Statistical Sciences, Sapienza University of Rome, Rome 00185, Italy; Department of Statistical Sciences, Sapienza University of Rome, Rome 00185, Italy; Dept. of Translational and Precision Medicine, Sapienza University of Rome, Rome 00161, Italy; Dept. of Translational and Precision Medicine, Sapienza University of Rome, Rome 00161, Italy

**Keywords:** microcarcinoma, papillary thyroid carcinoma, thyroid cancer, predictors, outcome

## Abstract

**Context:**

The risk of recurrence of papillary thyroid carcinoma (PTC) smaller than 1 cm (microPTC) is low. Predictors of disease persistence in microPTC are still unclear.

**Objective:**

To compare the clinical and pathological characteristics of microPTCs with macrocarcinomas (PTC > 1 cm), identifying the predictors of biochemical and structural incomplete response 1 year after initial treatment in microPTC.

**Methods:**

We included patients consecutively enrolled in the Italian Thyroid Cancer Observatory (NCT04031339), and selected patients with a histological diagnosis of PTC for whom complete pathological, clinical, treatment information, and results at the 1-year follow-up visits were available.

**Results:**

Among 5038 patients in the cohort, 2345 (46.5%) had a microPTC. Patients with microPTCs had tumors with more indolent pathological features: only 3% of patients were classified as high risk according to the American Thyroid Association (ATA) risk stratification system for persistent or recurrent disease and 1% had distant metastases at diagnosis. MicroPTCs had a significantly better outcome: only 5% had a biochemical response and 2.3% a structural incomplete (SIR) response. Distant metastases at diagnosis were the best predictor of SIR in microPTCs (OR 5.13, 95% CI 1.11-23.73, *P* = .04). In a subgroup of 925 patients treated by total thyroidectomy and radioiodine treatment, the best predictor of SIR was the ATA high risk (OR 5.47, 95% CI 1.42-21.04, *P* = .01).

**Conclusion:**

Our study confirms the favorable initial outcome of microPTC in a large series. We demonstrate that the ATA risk classification is reliable in predicting biochemical and structural persistence in patients with microPTC. Distant metastases, although rare, remain the best predictor of structural persistence at 1-year follow-up. These findings underscore the importance of tailored management strategies based on comprehensive risk stratification, rather than solely on tumor size.

Papillary thyroid carcinoma (PTC) is clinically defined as microcarcinoma (microPTC) if its dimensions are smaller than or equal to 1 cm in the greatest diameter. The incidence of PTC has increased worldwide since the early 1990s, mainly due to the rise in the detection of small, incidental, and often clinically silent tumors during neck imaging performed for unrelated medical issues (1). Today, in clinical practice, tumor size is often the primary criterion for guiding many treatment decisions, such as the extent of initial surgery or the need for ablative treatment with radioactive iodine (2, 3).

The dramatic rise in diagnoses of differentiated thyroid carcinoma necessitates a deeper understanding of the clinical and pathological features that may elevate the risk of recurrence or affect disease-free survival, particularly in cases of microPTC. Although the overall risk of recurrence in microPTC is low, ranging from 1% to 5% (4), there are instances where aggressive tumor behavior is observed despite small size. Therefore, establishing robust predictive factors for biochemical and structural recurrence is essential. This knowledge would allow a better selection of patients who can be treated with lobectomy rather than thyroidectomy or even without surgery, with subsequent monitoring through with active surveillance (5). Among these characteristics, the clinical evidence of suspicious lymph nodes in the lateral cervical compartments at initial diagnosis increases the risk of recurrence (4), as well as male sex, younger age (6, 7), and BRAF^V600E^ mutation (8). Incidental microPTCs have a lower recurrence rate than nonincidental microPTCs (9). Considering histological features, a retrospective multicenter study reported that neck lymph node involvement, tumor stage, and aggressive histology are independent predictors of persistent/recurrent disease (6). Additionally, the relationship between aggressive PTC variants and a poorer outcome has been documented (10). Conflicting results have been produced regarding microscopic extrathyroidal extension (4, 11), whose prognostic role remains to be clarified. Retrospective data on large patient cohorts also suggest a possible role of multifocality (12), which may be linked to lymph node metastasis rather than recurrence (13, 14).

This multicenter prospective study aims to describe the clinical and histological characteristics of microPTCs in comparison with macrocarcinomas (PTC > 1 cm), and to identify the factors predicting biochemical incomplete response (BIR) and/or structural incomplete response (SIR) 1 year after initial treatment. By addressing these objectives, we aim to provide insights into the postsurgical management and treatment strategies for patients diagnosed with microPTC.

## Materials and Methods

**Figure 1.**
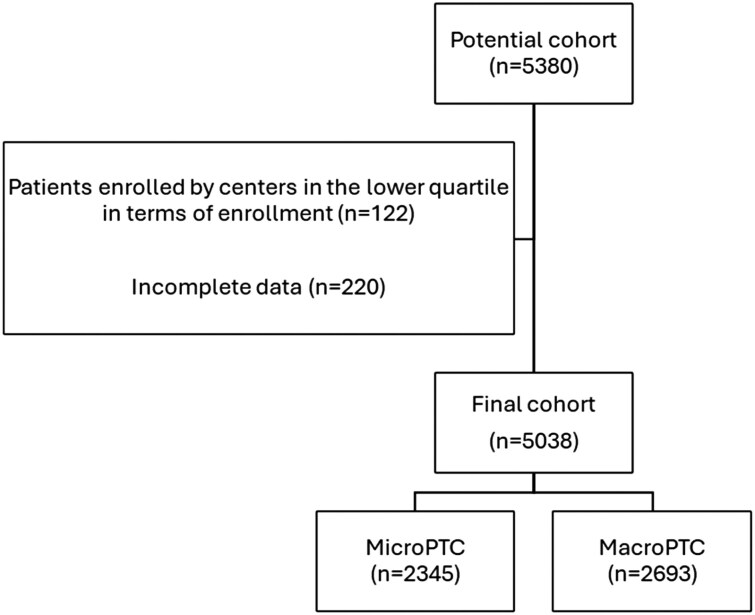
Flowchart of the participants.

The Italian Thyroid Cancer Observatory (ITCO), a web-based database, was started in 2013. The dataset includes prospectively collected data from over 12 000 patients with histologically confirmed diagnoses of differentiated, poorly differentiated, anaplastic, and medullary thyroid cancer from 51 participating thyroid cancer centers, with at least 1 first follow-up visit performed 6 to 18 months after surgery. The registered prospective study (ClinicalTrials.gov NCT04031339) has received ethics approval from the Coordinating Center Ethics Committee (Sapienza University of Rome, ref. 3366) and the individual ethics committees associated with each participating center. Cases are included in the database at the time of initial treatment by the reporting ITCO center, or when the patient begins follow-up in the reporting center within 12 months after undergoing initial treatment in a non-ITCO center, after signing the informed consent form. We have already reported assessments of the baseline risk estimates (15) on the first patients reaching early follow-up evaluations, and of the role of specific features (16-18). For this study, we reviewed all records available in the ITCO database and selected consecutive cases that satisfied the following criteria: (1) histological diagnosis of PTC; (2) availability of the results at 1-year follow-up visits (after initial treatment, ±6 months), including all data required to classify the treatment response, and (3) patients enrolled in clinical centers that are above the first quartile in terms of case frequency. After having applied the aforementioned inclusion criteria, 5038 patients were included (Fig. 1). The response to initial therapy was evaluated based on the collected clinical data, including imaging findings (cervical ultrasound for all patients and radioiodine scintigraphy for selected individuals) and serum thyroglobulin and thyroglobulin antibody levels. Additional imaging studies were performed at the clinicians’ discretion. The results were classified as per the 2015 American Thyroid Association (ATA) guidelines for patients who underwent thyroidectomy followed by radioactive iodine treatment (RAIT) (2) and as recommended by the 2019 European Society for Medical Oncology (3). Aggressive variants of PTC included tall cell, solid, hobnail, columnar, trabecular, and sclerosing variants. Vascular invasion was distinguished from lymphovascular invasion and defined as the presence of tumor cells within the lumen of a blood vessel.

### Statistical Analysis

In our descriptive analysis, continuous variables were summarized using medians with interquartile ranges (IQR), while categorical variables were described in terms of frequency counts and corresponding percentages. To evaluate significant association in contingency tables, we used the chi-squared test. The Welch t-test compares means of continuous variables in 2 different groups, therefore it was used to assess the association between continuous variables in different groups.

The response to treatment was first analyzed as a categorical variable with 4 ordered levels (structural incomplete, biochemical incomplete, indeterminate, and excellent). Furthermore, we analyzed treatment response as a binary variable classified as either an excellent/indeterminate or SIR/BIR. Moreover, we considered the SIR vs the other 3 categories. We used a mixed logistic model with a center-specific random intercept to evaluate possible predictors of these responses. The variables included in the model were sex, preoperative cytology, surgical approach, use of RAIT, neck dissection, PTC subtype, tumor foci, lymph node metastasis status, presence of distant metastases, and ATA risk. Given the hierarchical structure of the data, with patients nested within clinical centers, the center-specific intercept is used to summarize unobserved center-specific characteristics. The integral defining the likelihood was approximated via the Laplace approximation. The models were also implemented by stratifying patients by type of treatment. This analysis was performed using the R library ordinal (19).

## Results

### Clinical and Pathological Profiles of Included Patients

The clinical and pathological characteristics of the 5038 patients with PTC enrolled in the study are summarized in Table 1. Among them, 2345 (46.5%) had a microPTC (tumors ≤1 cm), while 2693 (53.5%) had a macroPTC (tumors >1 cm). Female gender was predominant (75%) and more frequent in the microPTC group (78% compared to 71% in the macroPTC cohort, *P* < .001). The median age at diagnosis was 49 years (IQR 39-59 years) and patients with a microPTC were slightly younger (median age 48 vs 50 years, *P* < .001). The rate of incidental findings among microPTC was higher, while suspicious or malignant preoperative cytology was more prevalent in patients with macroPTC (46.6% vs 64%, *P* < .001). Total thyroidectomy was performed in 92% of cases, while lobectomy and near-total thyroidectomy were each conducted in 4% of patients; 56.6% of patients received RAIT following total thyroidectomy. Neck dissection was required in 40% of patients, with about 13% requiring lateral neck lymphadenectomy; as expected, lobectomy was more frequently performed in microPTC, while RAIT and neck lymphadenectomy (both central and lateral) were more common in the macroPTC cohort (*P* < .001). Histologically, half of PTC cases were classified as classic, 30% were follicular variants, and aggressive variants were reported in 331/5038 (6.6%) cases; aggressive variants were less prevalent in the microPTC group (115/2345, 4.9%, *P* < .001) than in the macroPTC group (216/2693, 8%). MicroPTCs were more often unilateral (59.8% vs 55%, *P* < .001) and over three-quarters were intrathyroidal (76.6% vs 62.2%, *P* < .001) but, interestingly, up to 22% had microscopic and 2% macroscopic extrathyroidal extension. Distant metastases were reported in 122 patients (2.4%) and were rarer in microPTCs (1.1% vs 3.6%, respectively, both *P* < .01). According to the ATA risk classification, 2539 (50.4%) tumors of the entire cohort were categorized as low-risk, 2197 (43.6%) as intermediate-risk, and 302 (6%) as high-risk. MicroPTC fell more often in the ATA low-risk class; in particular, microPTC and macroPTC were, respectively, low-risk tumors in 61.8% and 40.5%, intermediate-risk tumors in 34.8% and 51.2%, and high-risk tumors in 3.4% vs 8.3% of cases (*P* < .001).

**Table 1.** Clinical and pathological characteristics of the study cohort and according to PTC size: microPTC compared to macroPTC

		All cohort	MicroPTC	MacroPTC	*P*
n (%)		5038	2345 (46.5)	2693 (53.5)	
Gender, n (%)	F	3748 (74.4)	1834 (78.2)	1914 (71.1)	<.001
Age, years	Median IQR	49 39-59	48 37-59	50 40-60	<.001
Preoperative cytology, n (%)*^a^*	Not done/not specified	794 (15.8)	520 (22.2)	274 (10.2)	<.001
	Nondiagnostic	49 (1)	30 (1.3)	19 (0.7)	
	Benign	260 (5.2)	166 (7.1)	94 (3.5)	
	Indeterminate	1118 (22.2)	536 (22.9)	582 (21.6)	
	Suspicious/Malignant	2817 (55.9)	1093 (46.6)	1724 (64)	
Surgical approach, n (%)	Lobectomy	202 (4)	131 (5.6)	71 (2.6)	<.001
	Near total thyroidectomy	197 (3.9)	86 (3.7)	111 (4.1)	
	Total thyroidectomy	4639 (92.1)	2128 (90.8)	2511 (93.2)	
RAIT, n (%)	Performed	2850 (56.6)	925 (39.5)	1925 (71.5)	<.001
Neck dissection, n (%)	Not performed	3069 (60.9)	1598 (68.1)	1471 (54.6)	<.001
	CC	1328 (26.4)	496 (21.2)	832 (30.9)	
	LC	107 (2.1)	55 (2.4)	52 (1.9)	
	CC + LC	534 (10.6)	196 (8.4)	338 (12.6)	
PTC variant, n (%)	Unknown	253 (5)	145 (6.2)	108 (4)	<.001
	Classic	2659 (52.8)	1287 (54.9)	1372 (51)	
	Follicular	1459 (29)	662 (28.2)	797 (29.6)	
	Others	336 (6.7)	136 (5.8)	200 (7.4)	
	Tall cell	195 (3.9)	73 (3.1)	122 (4.5)	
	Other aggress. variants*^b^*	136 (2.7)	42 (1.8)	94 (3.5)	
Tumor size, mm	Median IQR	11 7-18	7 4.5-9	17 13-25	<.001
Tumor foci, n (%) (n = 4953)	Unilateral	2834 (57.2)	1382 (59.8)	1452 (55)	<.001
	Multifocal, not specified	61 (1.2)	29 (1.3)	32 (1.2)	
	Multifocal, unilateral	667 (13.5)	341 (14.7)	326 (12.3)	
	Multifocal, bilateral	1391 (28.1)	560 (24.2)	831 (31.5)	
Extrathyroidal extension, n (%) (n = 5023)	None	3460 (68.9)	1793 (76.6)	1667 (62.2)	<.001
	Microscopic	1412 (28.1)	507 (21.6)	905 (33.7)	
	Macroscopic (T4a)	143 (2.8)	39 (1.7)	104 (3.9)	
	Macroscopic (T4b)	8 (0.2)	2 (0.1)	6 (0.2)	
Lymph node metastases, n (%) (n = 5031)	NX	1298 (25.8)	752 (32.2)	546 (20.3)	<.001
	N0	2457 (48.8)	1157 (49.5)	1300 (48.3)	
	N1a	726 (14.4)	224 (9.6)	502 (18.7)	
	N1b	550 (10.9)	208 (8.9)	342 (12.7)	
Vascular invasion, n (%) (n = 4828)	Yes	800 (16.5)	175 (7.8)	625 (24.2)	<.001
Distant metastases, n (%)	Yes	122 (2.4)	25 (1.1)	97 (3.6)	<.001
ATA risk, n (%)	Low	2539 (50.4)	1449 (61.8)	1090 (40.5)	<.001
	Intermediate	2197 (43.6)	817 (34.8)	1380 (51.2)	
	High	302 (6)	79 (3.4)	223 (8.3)	

Please note that when the data was not available in the whole cohort, the number of cases included is reported in brackets in the first column.

Abbreviations: ATA, American Thyroid Association; CC, central compartment; F, female; IQR, interquartile range; LC, lateral neck compartment; RAIT, radioactive iodine treatment; PTC, papillary thyroid cancer; TT, total thyroidectomy.

^a^Preoperative cytology refers to the results of fine needle aspiration if performed.

^b^Aggressive variants include solid, insular, columnar-cell, hobnail-cell, and sclerosing histologic subtypes.

### Assessment of Outcomes and Structural Persistence Characterization

One year after the initial treatment, an excellent or indeterminate response was recorded in 4543 patients (90.1% of the whole cohort), with BIR in 303 patients (6%) and SIR in 192 patients (3.8%). MicroPTCs were more frequently characterized by an excellent or indeterminate response at the 1-year follow-up visit (92.7% vs 88% observed in patients with macroPTCs). Both BIR and SIR were more prevalent in macroPTCs (6.9 and 5.1%, respectively) than in microPTCs (5% and 2.3%, respectively, *P* < .001) (Table 2).

**Table 2.** Treatment response at 1-year evaluation in microPTCs compared to macroPTCs (n = 5038)

Treatment response	All, n (%)	MicroPTC, n (%)	MacroPTC, n (%)	*P*
Excellent	2491 (49.4)	1204 (51.3)	1287 (47.8)	<.001
Indeterminate	2052 (40.7)	970 (41.4)	1082 (40.2)	
Biochemical incomplete	303 (6)	117 (5)	186 (6.9)	
Structural incomplete	192 (3.8)	54 (2.3)	138 (5.1)	

In microPTC and macroPTC, SIR was determined by the presence of suspicious tissue in thyroid bed in 6/54 (0.25% of microPTC) and 23/138 patients (0.85% of macroPTC), suspicious lymph nodes in 38/54 (1.6% of microPTC) and 86/138 patients (3.2% of macroPTC), suspicious distant metastases in 12/54 (0.5% of microPTC) and 38/138 patients (1.4% of macroPTC), respectively. The presence of lymph nodes with a suspicious appearance was observed in the central compartment in 15/38 (39.5%) and 24/86 patients (27.9%), in the lateral compartment in 20/38 (52.6%) and 52/86 patients (60.5%), and in both the central and the lateral compartment in 3/38 (7.9%) and 7/86 patients (8.1%), in microPTC and macroPTC, respectively.

### Predictors of Evidence of Disease in microPTC Tumors

Logistic regression analysis identified several parameters associated with evidence of disease (both biochemical and structural) at the 1-year follow-up visit. These included the presence of central compartment lymph node metastases (OR of 2.12, 95% CI 1.18-3.80, *P* = .01), the presence of distant metastases (OR 3.7, 95% CI 1.1-12.46, *P* = .03), the use of RAIT (OR 2.04, 95% CI 1.28-3.26, *P* < .001), and near total thyroidectomy (OR 4.51, 95% CI 1.76-11.53, *P* < .001).

For structural evidence of disease (compared to excellent, indeterminate, and biochemical response) significant predictors included distant metastases at diagnosis (OR 5.13, 95% CI 1.11-23.73, *P* = .04) and the use of RAIT (OR 2.23, 95% CI 0.99-5.05, *P* = .05) (Table 3).

**Table 3.** Predictors of evidence of disease in microPTCs, after logistic regression analysis (n = 2345)

Outcome	Variables	OR (95% CI)	*P*
BIR + SIR (vs IND + ER)	Female sex	1.04 (0.7-1.54)	.86
	ATA intermediate risk	1.1 (0.66-1.83)	.71
	ATA high risk	1.57 (0.61-4.09)	.35
	Follicular variant	0.73 (0.47-1.13)	.16
	Other nonaggressive variants	1.22 (0.73-2.02)	.45
	Aggressive variants	0.85 (0.4-1.79)	.66
	Lobectomy	1.25 (0.54-2.94)	.6
	Near total thyroidectomy	**4.51** (**1.76-11.53)**	**<**.**001**
	RAIT	**2.04** (**1.28-3.26)**	**<**.**001**
	Not done/not specified cytology	1.0 (0.48-2.07)	1.0
	Non diagnostic cytology	1.06 (0.23-4.98)	.94
	Indeterminate cytology	0.84 (0.47-1.48)	.55
	Suspicious cytology	1.06 (0.61-1.84)	.83
	Malignant cytology	1.06 (0.63-1.8)	.82
	Multifocal, not specified	0.96 (0.21-4.45)	.96
	Multifocal, unilateral	1.21 (0.75-1.96)	.43
	Multifocal, bilateral	1.24 (0.83-1.85)	.28
	Lateral compartment neck dissection	1.12 (0.42-2.98)	.82
	NX (vs N0)	1.38 (0.87-2.18)	.17
	N1a	**2.12** (**1.18-3.8)**	.**01**
	N1b	1.88 (0.64-5.51)	.25
	Distant metastases	**3.7** (**1.1-12.46)**	.**03**
SIR (vs BIR + IND + ER)	Female sex	0.59 (0.32-1.09)	.09
	ATA intermediate risk	1.18 (0.48-2.86)	.72
	ATA high risk	2.01 (0.5-8.16)	.33
	Follicular variant	0.94 (0.46-1.93)	.87
	Other nonaggressive variants	0.63 (0.21-1.9)	.41
	Aggressive variants	1.32 (0.46-3.79)	.6
	Lobectomy	1.47 (0.31-6.86)	.63
	Near total thyroidectomy	0.72 (0.08-6.18)	.77
	RAIT	**2.23** (**0.99-5.05)**	.**05**
	Not done/not specified cytology	1.5 (0.45-5.05)	.51
	Non diagnostic cytology	NA*^a^*	ns*^a^*
	Indeterminate cytology	0.71 (0.23-2.19)	.56
	Suspicious cytology	1.31 (0.49-3.46)	.59
	Malignant cytology	1.55 (0.61-3.91)	.35
	Multifocal, not specified	NA*^a^*	ns*^a^*
	Multifocal, unilateral	1.16 (0.52-2.58)	.72
	Multifocal, bilateral	1.1 (0.56-2.13)	.79
	Lateral compartment neck dissection	1.18 (0.26-5.43)	.83
	NX (vs N0)	1.72 (0.77-3.82)	.18
	N1a	1.81 (0.67-4.88)	.24
	N1b	2.04 (0.38-10.91)	.4
	Distant metastases	**5.13** (**1.11-23.73)**	.**04**

Bold values highlight statistically significant features. Abbreviations: ATA, American Thyroid Association; BIR, biochemical incomplete response; ER, excellent response; IND, indeterminate response; NA, not assessable; PTC, papillary thyroid cancer; RAIT, radioactive iodine treatment; SIR, structural incomplete response.

^a^Due to insufficient information in the model to estimate the standard error, the CI cannot be determined.

Further analysis of a subgroup of 925 patients who underwent total thyroidectomy and RAIT, excluding distant metastasis variable due to its rarity in the microPTC group, revealed that predictors of biochemical and structural evidence of disease included ATA high-risk classification (OR 3.67, 95% CI 1.52-8.84, *P* < .001), detection of central neck compartment lymph node metastases at initial surgery (OR 2.69, 95% CI 1.31-5.53, *P* = .01), or a not performing neck lymphadenectomy (OR 2.47, 95% CI 1.21-5.03, *P* = .01). The sole predictor of structural evidence of disease in this subgroup was the ATA high risk (OR 5.47, 95% CI 1.42-21.04, *P* = .01) (Table 4).

**Table 4.** Predictors of evidence of microPTCs treated by total thyroidectomy and radioactive iodine treatment, after logistic regression analysis (n = 925)

Outcome	Variables	OR (95% CI)	*P*
BIR + SIR (vs IND + ER)	Female sex	0.91 (0.55-1.52)	.73
	ATA intermediate risk	1.46 (0.71-3.0)	.31
	ATA high risk	**3.67** (**1.52-8.84)**	**<**.**001**
	Follicular variant	0.81 (0.44-1.47)	.48
	Other nonaggressive variants	1.69 (0.89-3.23)	.11
	Aggressive variants	0.99 (0.43-2.26)	.98
	Not done/not specified cytology	0.99 (0.39-2.55)	.98
	Non diagnostic cytology	1.12 (0.11-11.83)	.93
	Indeterminate cytology	0.65 (0.26-1.65)	.37
	Suspicious cytology	0.95 (0.44-2.05)	.89
	Malignant cytology	0.87 (0.42-1.82)	.72
	Multifocal, not specified	0.95 (0.19-4.87)	.95
	Multifocal, unilateral	0.91 (0.47-1.78)	.79
	Multifocal, bilateral	1.17 (0.71-1.91)	.54
	Lateral compartment neck dissection	1.41 (0.48-4.17)	.53
	NX (vs N0)	**2.47** (**1.21-5.03)**	.**01**
	N1a	**2.69** (**1.31-5.53)**	.**01**
	N1b	1.73 (0.52-5.78)	.38
SIR (vs BIR + IND + ER)	Female sex	0.51 (0.25-1.06)	.07
	ATA intermediate risk	1.39 (0.41-4.74)	.6
	ATA high risk	**5.47** (**1.42-21.04)**	.**01**
	Follicular variant	0.59 (0.21-1.63)	.3
	Other nonaggressive variants	0.96 (0.31-3.0)	.95
	Aggressive variants	1.42 (0.48-4.2)	.53
	Not done/not specified cytology	0.95 (0.23-3.97)	.98
	Non diagnostic cytology	NA*^a^*	ns*^a^*
	Indeterminate cytology	0.31 (0.05-1.73)	.18
	Suspicious cytology	0.83 (0.25-2.71)	.76
	Malignant cytology	0.96 (0.32-2.88)	.94
	Multifocal, not specified	NA*^a^*	ns*^a^*
	Multifocal, unilateral	1.0 (0.37-2.72)	1.0
	Multifocal, bilateral	0.87 (0.5-2.28)	1.07
	Lateral compartment neck dissection	1.39 (0.27-7.25)	.7
	NX (vs N0)	1.68 (0.53-5.33)	.38
	N1a	2.27 (0.72-7.16)	.16
	N1b	1.76 (0.28-11.05)	.55

Bold values highlight statistically significant features. Abbreviations: ATA, American Thyroid Association; BIR, biochemical incomplete response; ER, excellent response; IND, indeterminate response; NA, not assessable; PTC, papillary thyroid cancer; RAIT, radioactive iodine treatment; SIR, structural incomplete response.

^a^Due to insufficient information in the model to estimate the standard error, the confidence interval cannot be determined.

Patients with aggressive variants in microPTC exhibited a nonsignificant risk of structural disease (OR 1.32, 95% CI 0.46-3.79, *P* = NS), but were treated more aggressively: RAIT administered to 85 out 115 (73.9%) patients with aggressive variants, compared to 516 out 1287 (40.1%) PTCs with classic histology and 216 out 662 (32.6%) with follicular variants. However, also in the subgroup of patients submitted to total thyroidectomy and RAIT, no significant risk of structural persistence was recorded for aggressive variants (OR 1.42, 95% CI 0.62-3.23, *P* = NS).

## Discussion

In this study, with more than 5000 prospectively collected PTCs, we evaluated the outcome of microPTC and the potential predictors of persistent disease. As expected, microPTCs, compared to macroPTCs, had clinical and histological features typically associated with a favorable prognosis. Specifically, microPTCs were more often diagnosed in younger female patients, and the tumors were predominantly unilateral, intrathyroidal, without vascular invasion, and had lower rates of lymph node and distant metastases. Notably, approximately one-third of microPTC cases were identified incidentally, a significant proportion were classified as ATA low-risk tumors, with high-risk tumors accounting for only 3% of cases. Overall, we observed a good response 1 year after initial treatment for microPTCs, with over 90% of patients achieving excellent or indeterminate responses. Even though the 2009 and the updated 2015 ATA guidelines proposed thyroid lobectomy for microPTCs (2), total thyroidectomy was still preferred in more than 90% of cases in our Italian cohort. Moreover, approximately 40% of these patients underwent RAI treatment. A delay in the translation of guideline recommendations into clinical practice has been noted, and is influenced by factors such as physician knowledge and attitudes and patient preferences (20). Similar discrepancies between ATA guidelines and practice have been observed in the United States. For instance, a recent study using Surveillance, Epidemiology, and End Results cancer data reported that lobectomy was performed in only 25% to 30% of microPTCs, with lobectomy rate increasing more slowly than expected after the updated ATA guidelines were introduced (21). Given the established safety of lobectomy for thyroid cancer (22), we anticipate a future rise in lobectomy rates accompanied by a decline in RAI ablation use. The ITCO study offers a valuable resource to track real-world clinical practices and evaluate the impact of evidence-based guidelines. Nevertheless, concerted efforts are essential to facilitate the effective implementation of thyroid cancer guidelines in clinical settings.

Despite the overall good prognosis of microPTC, we noted that 38.2% of cases were classified as intermediate or high risk, and 5% of patients experienced BIR and 2.3% had SIR with the same timeframe. This prompted us to identify predictors of disease evidence in microPTCs, to facilitate tailored treatment strategies, allowing for a more conservative approach in the majority of patients with indolent tumors, while reserving a more aggressive management for the minority with more concerning features. The presence of distant metastases at diagnosis emerged as the strongest single predictor of structural persistence, albeit this finding was rare, occurring in only 1% of microPTC cases. Previous studies have identified older age at diagnosis, extrathyroidal extension, and lymph node metastases are independent risk factors for distant metastases in microPTC (23). However, these variables failed to be predictive of SIR in our study. Therefore, only the finding of distant metastasis should prompt clinicians to a different attitude towards patients with microPTCs.

The use of RAIT was a weak predictor of structural persistence but gained significance when both SIR and BIR are considered together. This aligns with the understanding RAIT is often reserved for patients deemed at higher risk based on clinical judgment. Additionally, central neck compartment lymph node and distant metastases were associated with SIR and BIR. Finally, near-total thyroidectomy was associated with BIR, presumably due to remaining thyroid tissue after surgery.

To further refine our analysis, we examined a subgroup of 925 patients who underwent total thyroidectomy and RAIT, thereby minimizing treatment variability. In this cohort we excluded distant metastases from this model due to the low frequency of the event, which made the model unreliable. Within this subgroup, the ATA high-risk classification was the sole predictor of SIR. Predictors of BIR and SIR were again ATA high-risk, central neck lymph node metastases, and absence of neck dissection. The application of the 2015 ATA risk stratification system to determine the risk of recurrence (2) proved beneficial in discerning which patients might require a more aggressive management. While the efficacy of this system in microPTC has been suggested, it has not been robustly validated. Indeed, previous studies have shown mixed results regarding the predictive value of ATA classification in microPTC, highlighting the need for further investigation. Thus, a single-center retrospective study on 357 subjects found no differences between low and intermediate-risk microPTC, while patients with high-risk microPTC have an increased risk of recurrence compared to the others (24). In another retrospective study with a smaller sample size (n = 67), the ATA risk stratification system showed high specificity and negative predictive value but relatively low sensitivity and positive predictive value, probably because of the very low number of recurrences in the small cohort (25). However, our data strongly indicate that ATA risk has a preponderant prognostic role also in microPTC.

In our cohort, lymph node metastases were diagnosed in less than 20% of patients with microPTC. In previous studies, particularly when prophylactic central neck lymph node dissection was performed, this percentage exceeded 40% (26-28). It can thus be postulated that the higher BIR rate observed 1 year after initial treatment, both in patients with central neck compartment metastases and in patients that did not undergo lymph node neck dissection, may be attributed to the presence of undiscovered lymph node micrometastases. While our study did not extend the follow-up on these patients beyond 1 year, the impact of micrometastases on disease-free survival is generally considered minimal (2, 3, 28). Notably, in our study, the risk of SIR was not increased in the presence of central compartment lymph node metastases, which influenced only BIR at the 1-year evaluation following initial treatment.

Finally, 5% of our microPTCs had aggressive variants, particularly tall cell subtypes. The rate of aggressive variants is variable among studies and reported to range from 0.5% to 6% (9, 10, 29). Aggressive variants in our study were treated more aggressively, particularly considering the use of RAIT. However, in the subgroup of microPTCs treated with total thyroidectomy and RAIT, aggressive variants were not able to predict disease persistence (both biochemical and structural), even though the risk was slightly higher in these cases. A previous study reported aggressive histology as a predictor of persistence or recurrence (9). Another study demonstrated that aggressive subtypes (tall cell, solid, hobnail, columnar cell, diffuse sclerosing) have worse behavior even among small tumors (10). In the recently published fifth edition of the World Health Organization Classification of Endocrine and Neuroendocrine Tumors, microPTC is not considered as a separate PTC subtype (30), underlying the importance of defining the subtype regardless of tumor size. In our study, despite the slightly higher risk of persistence of microPTC with aggressive variants, other factors, particularly the ATA risk at diagnosis, play an even more important role in predicting the outcome of these tumors and better help in selecting the microPTC deserving particular attention.

The main limitation of our study is the lack of follow-up after 1 year from the initial treatment. However, because of the good response to treatment of these tumors, the results of this study can reassure us about the outcome of the majority of these cancers, which are effectively in remission after 1 year. Nevertheless, a prolonged follow-up period is necessary to comprehensively assess the risk of recurrence in this group of PTCs. An evaluation with a longer follow-up will be performed to assess the outcome of the minority of tumors not cured after 1 year. The other limitation pertains to aggressive variants: the number of aggressive variants in microPTC included in this study is one of the largest published so far, but their reporting was not mandatory in the context of microPTCs in previous editions of World Health Organization manuals. It is possible that some aggressive features were overlooked in small tumors in previous years. Finally, the pathological reports and features analyzed in our study reflect assessments made by pathologists in a real-world observational setting rather than a controlled research environment. This approach has its own inherent strengths and limitations. Nevertheless, our findings are consistent with rates observed in both clinical and interventional studies. A recent study, which employed advanced methodologies including immunohistochemistry for detecting vascular invasion (31), reported a rate aligned to that observed in our study.

## Conclusions

Our results confirm, in a very large series, the favorable outcome 1 year after surgery associated with microPTC. In patients treated by total thyroidectomy and RAIT, the ATA risk classification system is reliable in predicting both biochemical and structural persistence and appears to be a valid tool for selecting patients who deserve particular attention and potential further treatments, independently from the size of the tumors.

## Data Availability

The data presented in this study are available upon reasonable request from the corresponding author. The data are not publicly available due to restrictions.

## Clinical Trial Information

Clinicaltrial.gov registration number: NCT04031339 (registered June 25, 2019).

## Abbreviations

BIR: biochemical incomplete response
PTC: papillary thyroid carcinoma
RAIT: radioactive iodine treatment
SIR: structural incomplete response
